# A Case of Postpartum Obsessive-Compulsive Disorder in a First-Time Father

**DOI:** 10.7759/cureus.54547

**Published:** 2024-02-20

**Authors:** Kevin W Chen, Luke Schultz, Neil Hughes

**Affiliations:** 1 Psychiatry, Western Michigan University Homer Stryker M.D. School of Medicine, Kalamazoo, USA; 2 Internal Medicine, Western Michigan University Homer Stryker M.D. School of Medicine, Kalamazoo, USA

**Keywords:** perinatal ocd, postpartum anxiety, paternal postpartum depression, postpartum ocd, postpartum mental health, ocd/ anxiety disorders

## Abstract

Obsessive-Compulsive Disorder (OCD) is a well-recognized psychiatric condition characterized by distressing obsessions and compulsions. While the perinatal period is a known trigger for OCD in women, less attention has been given to its occurrence in men, particularly new fathers. This case report examines the unique presentation of postpartum-onset OCD (ppOCD) in a first-time father.

A 33-year-old father presented eight months after the birth of his first child with distressing intrusive thoughts related to harming his eight-month-old daughter. These thoughts were ego-dystonic, causing significant distress, and led to a rapid deterioration in his mental health. Intrusive thoughts included a desire to leave his daughter in a busy street and place her in a hot oven. The patient became severely depressed, experienced significant weight loss, and was unable to perform daily activities of living. He repeatedly denied any intent to act on these thoughts. Following a visit to the ED, the patient was admitted to a psychiatric facility and started on escitalopram and aripiprazole. Approximately one month post-discharge, the patient reported significant symptom improvement, and after two months, his symptoms were well-controlled. He was successfully tapered off aripiprazole due to remission of symptoms and adverse effects.

This case report highlights the need for greater awareness and screening of ppOCD in both men and women during the perinatal period. Utilizing existing screening tools and well-established pharmacological treatments for OCD can significantly improve the recognition and management of this distressing disorder in fathers, ultimately improving their quality of life and that of their families. Further research is needed to better understand the prevalence and specific management of male ppOCD.

## Introduction

The 5th edition of the Diagnostic and Statistical Manual of Mental Disorders (DSM-5) defines obsessions as involuntary and persistent thoughts or urges that in most individuals cause marked anxiety or distress. Compulsions are defined as behaviors or mental acts aimed to prevent or reduce anxiety resulting from obsessions. The diagnosis of Obsessive-Compulsive Disorder (OCD) requires the presence of obsessions, compulsions, or both that cause clinically significant distress or impairment in daily functioning [1]. In women, the perinatal period has been a well-recognized trigger of OCD since the mid to late 1900s. While perinatal onset is not currently a formal specifier in the DSM-5, some suggest making it so [2,3]. Obsessive symptoms are also frequently observed in perinatal women even without OCD. In a prospective cohort study, up to 37% of women demonstrated subclinical obsessions or compulsions at two weeks postpartum [4]. A particularly unique and shared feature of postpartum obsessions is intrusive thoughts of harming the baby [5,6]. It is important to note that these obsessions are ego-dystonic, thus causing distress. This differentiates postpartum-onset OCD (ppOCD) from brief psychotic disorder with postpartum onset (also known as postpartum psychosis), which presents as ego-syntonic desires to harm the newborn baby [1].

The lifetime prevalence of OCD is estimated to be 1.3%, with women being at 1.6 times higher risk than men [7]. It is logical that the majority of research on ppOCD centers around women. However, as early as 1999 it has been noted that new fathers demonstrate anxious intrusive thoughts associated with harm-avoidant behaviors which closely resemble OCD [8].

## Case presentation

A 33-year-old male with a past psychiatric history of depression in childhood and self-described "panic attacks" at age 18 presented to his primary care clinic with a chief complaint of 10 days of depressed mood and intrusive thoughts. The patient and his significant other had their first child eight months ago; since then, he reported being a “very caring father” and sharing childcare responsibilities, which was endorsed by his significant other. However, 10 days prior to presentation the patient experienced his first intrusive thought: while walking with his daughter in a stroller, he passed a busy crosswalk when he suddenly developed an intense desire to leave his daughter in the middle of the street and return home. He felt very fearful of acting on these urges and sat down on a nearby bench with his daughter until the intrusive thoughts eventually subsided. Notably, this episode coincided with the recent ending of the class he was teaching as a PhD student. In the days following the initial episode, he reported having increasingly intrusive thoughts which he describes as “spikes” in his brain centering around harming his daughter. Per the patient, the most distressing thought occurred a few days prior to the presentation. He was making breakfast with his daughter nearby when he had an intrusive thought to place his daughter into the hot oven. Having this thought was so distressing that it caused the patient to become severely depressed. Since then, he reported spending most of his days sleeping, not eating or drinking properly, and being unable to perform regular daily activities. He additionally avoided spending time alone with the baby due to fears of acting on these obsessive thoughts. The patient states that he has been unable to eat meat particularly because it reminds him of his “cooked daughter”. He reports that when he is not sleeping, he is doing significant research into his symptoms and has frequent rumination. He states the intrusive thoughts are upsetting because he loves his daughter very much and does not want to act on them. The patient repeatedly denied any plan, desire, or intent to harm himself, his child, or anyone else. 

During the interview with the patient, he was visibly anxious with a significantly dysphoric mood and congruent affect, crying repeatedly throughout the discussion. He was fully oriented with normal thought content and judgement, however at times having tangential thoughts requiring redirection. During the visit, he completed a nine-item Patient Health Questionnaire (PHQ-9) for which the score was 22. After the interview, the patient met with the on-site social worker who created a safety plan and directed the patient to a community behavioral health organization for assessment the next day. Additionally, the patient and the social worker contacted the significant other together to inform her of the plan. She was supportive and agreeable to the plan. At the conclusion of the visit, the patient reported feeling better and more optimistic about his recovery. He was future oriented and shared that he enjoyed teaching and wanted to be a good father. The patient agreed to laboratory workup including a blood count, comprehensive metabolic panel, and thyroid testing, which all returned unremarkable. The patient was prescribed 20 mg daily escitalopram and scheduled for a 2-week follow-up.

However, the next day, the patient presented to the emergency department (ED) due to increasingly intrusive thoughts. He reported that he initially felt better, but the thoughts continued to become more frequent and more difficult to “brush off”. The patient states he had a self-described panic attack which prompted him to come into the ED. He again denied any intent to harm himself or his daughter. He also denied any hallucinations, alcohol or drug use, or physical symptoms. Laboratory workup in the emergency department showed a negative urine drug screen. In the ED, the patient was willing to be voluntarily placed in an inpatient psychiatric facility. The patient was then transferred to a psychiatric facility where he was started on 5 mg aripiprazole per day as well as 20 mg escitalopram. He was discharged after 10 days in stable condition after it was determined he could safely continue treatment in an outpatient setting.

Approximately one month after discharge from the psychiatric facility, the patient returned to his primary care clinic for follow-up. At that time, he reported a significant improvement in his symptoms and denied intrusive thoughts for the past two weeks. He reports he has been able to spend time with his daughter and is able to take care of her without much distress. Two months after discharge, the patient stated that his symptoms are well controlled, and he has not had any additional intrusive thoughts. His PHQ-9 score at this time was 8. Due to medication adverse effects and the remission of his symptoms, his aripiprazole was reduced from 5 mg daily to 2 mg daily. Two weeks later, he reported continued remission of his symptoms and decreased adverse effects. His PHQ-9 remained stable, and the decision was made to discontinue aripiprazole completely. The patient has since been seen multiple times up to four months after the initial presentation without any reoccurrence of intrusive thoughts and well-controlled depressive symptoms.

## Discussion

Male ppOCD is a rarely reported phenomenon that presents with ego-dystonic intrusive thoughts identical to those seen in female ppOCD. To the best of our knowledge, there is only one other four-case series of paternal ppOCD that has been published to date (Table 1) [9]. Our case stands out from the previously reported cases in the timeline of symptom onset. The onset of intrusive thoughts in our patient abruptly started eight months after the birth of his baby, with rapid progression. Whereas in the four-case series previously published, all symptoms started within days after the birth of the child. This demonstrates that there remains much to be studied about the chronicity of male ppOCD. It suggests that a reasonable definition of ppOCD should include the onset of symptoms up to 12 months of birth, similar to postpartum depression [1].

**Table 1:** Details of previously published case series of paternal ppOCD Age, first-time father status, treatment modality, outcome, and resolution from the previously published four case series [9] in comparison to our patient. ppOCD: postpartum-onset obsessive-compulsive disorder; CBT: cognitive behavioral therapy; SSRIs: selective serotonin reuptake inhibitors

Case	Age	First Time Father?	Treatment	Outcome/Resolution of Symptoms
1	28	Yes	CBT	Good/Nearly Resolved
2	38	No (3rd child)	None	Good/Resolved
3	35	Yes	CBT	Good/Partially Resolved
4	40	Yes	CBT + SSRIs	Fair/Partially Resolved
Our patient	33	Yes	SSRIs + Antipsychotics	Good/Resolved

Currently, new or expecting mothers are not routinely screened for OCD. Our case highlights the need for awareness and screening for ppOCD, specifically in men. The two most commonly used screens for OCD are the Yale-Brown Obsessive-Compulsive Scale (YBOCS) and Compulsive Scale (DOCS), the latter of which was previously validated as a screening tool for perinatal OCD [10]. There has also been an effort to generate a perinatal-specific OCD screening tool, such as the Perinatal Obsessive-Compulsive Scale (POCS), to increase detection [11]. On initial presentation, our patient’s symptomatology would have resulted in positive screens using both DOCS and POCS, suggesting these scales would also function in detecting perinatal OCD in men. 

Due to the rare nature of male ppOCD, the prevalence remains uncertain. There exists one study aimed at detailing the epidemiology of male ppOCD, which demonstrated an approximately 5% prevalence in 726 fathers up to 60 days postpartum [12]. It should be noted that this study diagnosed OCD using the Mini-International Neuropsychiatric Interview, without the utilization of other tools to quantify symptom severity such as YBOCS or DOCS. Therefore, it remains a possibility that the reported 5% prevalence overestimates true ppOCD, instead detecting subclinical obsessions similar to those seen frequently in postpartum women without diagnosed OCD [4]. As such, the true prevalence of male ppOCD warrants investigation as it is likely underdiagnosed and under-reported due to the lack of recognition.

The primary first-line treatment for OCD has been widely accepted to be cognitive behavioral therapy (CBT), especially as a combination treatment with pharmacotherapy [13]. The case presented here, however, also demonstrates the variability in pharmacological approaches to treating ppOCD. Currently, there is a paucity of data on specific medication approaches for patients with OCD during the perinatal period. Preferred first-line medications for ppOCD are selective serotonin reuptake inhibitors (SSRIs) or clomipramine [14]. In the presented case, our patient was prescribed a dual therapy of an SSRI and an anti-psychotic, aripiprazole. There is one previous trial supporting the use of an anti-psychotic adjuvant alongside an SSRI in postpartum women with OCD [15]. Outside of the peri-natal period, there is strong evidence for the use of anti-psychotics for refractory cases of non-pregnancy-related OCD [16]. Our patient experienced full remission of intrusive thoughts with pharmacological treatment at four months and was able to discontinue the anti-psychotic medication. In the previously published case series, none of the cases reported the use of an anti-psychotic, and only one out of the four cases explicitly reported the complete absence of obsessions after treatment while the rest experienced only a reduction in symptoms. This suggests that in male cases of ppOCD, providers should refer to the normal pharmacological management of OCD, with good outcomes from CBT as well as anti-psychotic adjuvant medications.

## Conclusions

Paternal postpartum onset OCD is a rare, but likely underdiagnosed entity that has potentially profound effects on the quality of life of new fathers and their families. Several screening tools for OCD exist but are rarely, if ever, used for the identification of paternal ppOCD. There are also well-defined and effective pharmacologic treatment strategies for non-pregnancy OCD, which seem to have similar efficacy for patients with ppOCD. As there are existing screening tools as well as viable treatment options, it seems that recognition of ppOCD may be the largest barrier to effective treatment of this distressing disorder.
